# Tensions between Western and Indigenous worldviews in pharmacy education and practice: Part I

**DOI:** 10.1177/17151635231176250

**Published:** 2023-06-07

**Authors:** Jaris Swidrovich

**Affiliations:** Leslie Dan Faculty of Pharmacy, University of Toronto, Toronto, Ontario

## Introduction

Pharmacy, as an educational program and profession, is often not a friendly place for Indigenous learners and practitioners, particularly for those who see and understand the world through an Indigenous worldview. While Indigenous and Western knowledge systems may often be found in the same places and spaces, they remain as independent entities and often repel one another in the same way oil and water do when brought together. For each knowledge system to contribute to pharmacy education and practice, Western intellectual traditions may benefit from the use of an emulsifier, which might be a person or collective of persons raised with both Indigenous and Western knowledge systems, to bring the oil and water together as one. Through an Indigenous lens, the same concept may be visualized through metaphorical framing of each knowledge system as a strand in a braid of sweetgrass. Perhaps the best way forward is not to amalgamate each strand into a single large strand, but rather each should maintain its own composition while being interwoven together in a way that requires tension to become something bigger and stronger.^1^

This is the first in a series of 3 articles that will describe key differences and tensions that exist between Western intellectual traditions and Indigenous worldviews, both in general and within the context of pharmacy education and practice (Table 1).^2^ This article will describe the foundations of Indigenous knowledge systems and worldviews, and then the underpinnings of Western intellectual traditions, science and knowledge systems, and examine how they differ from one another. Given that Western knowledge is positioned as the foundation of pharmacy education and practice, this article will interrogate Western intellectual traditions in a similar way that Indigenous knowledge has been criticized, while respecting and emphasizing the importance of weaving multiple knowledge systems together for the greater good. Through a combination of both critical analyses of the literature and my own lived and living experiences as an Indigenous (Saulteaux First Nations) and Ukrainian person who completed pharmacy school and has spent more than 13 years as a practising pharmacist, these articles will analyze the foundations of 3 specific tensions in pharmacy education and practice between Western intellectual traditions and Indigenous worldview: 1) what constitutes evidence and truth (this article), 2) fragmentation and compartmentalization and 3) the lack of regard for land and spirituality.

**Table 1:** Differences between Western and Indigenous worldviews^2^

Western	Indigenous
Belief that ancestors were either mostly wrong or their ideas could be substantially improved upon	Belief that ancestors were right about most things
Belief in a reductionist and deterministic reality	Belief in an indivisible reality
Knowledge is situated in the material world and centred around the Gregorian calendar	Knowledge is situated within more expansive concepts of space, dimensions of reality and time
Human experience is separate from the natural world	Ways of being and science are constructed as part of the natural world
Tends to focus on only the observable dimension of reality	Belief in multiple dimensions of reality
Tends to focus on a scarcity of resources primarily driven by a conflation of want and need	Belief that there are sufficient resources to meet everyone’s needs
Focuses on the present and, to a lesser extent, on the future	Belief in expansive concepts of time in which the past, present and future are mutually influencing
Actions tend to focus on the present and both immediate and near future	Actions are focused on how they will impact the next 7 generations

## Indigenous knowledge systems and worldviews

The perspective about the nature of relationships between all life forms in creation has been called a worldview.^3^ For Indigenous Peoples, Indigenous worldviews have deep connections to tribe-specific creation stories and have lived and continue to live not through the written word but through oral history and storytelling.^4^ The validity of Indigenous knowledge is demarcated by its geographic and ecological setting for those who hold it and therefore is called “place-based” knowledge.^5-8^ Unfortunately, the term *Indigenous knowledge* itself is embedded within a Eurocentric epistemology and is therefore often replaced with a more appropriate phrase devised by Indigenous communities, such as “Indigenous ways of living” and “ways of being.”^5^ In fact, in the prevailing Eurocentric concept of school, *knowledge* (an accumulation of specific information, concepts and skills within a school subject) has no direct translation into most Indigenous languages because the Eurocentric concept of knowledge is largely foreign to most Indigenous worldviews.^5^ The best English expression for what Indigenous Peoples express as knowledge is “ways of living,” for which the word *learn* means “coming to knowing,”^9^ a concept closely related to Dewey’s^10^ participatory learning. “Knowledge is not a commodity that can be possessed or controlled by educational institutions, but is a living process to be absorbed and understood.”^11^

**summary**

- There are significant differences between Indigenous and Western worldviews.
- Each worldview is valid, important and critical in pharmacy education and practice.
- Learning about and appreciating the tensions between Indigenous and Western worldviews can improve the educational and practice experiences of Indigenous pharmacy professionals and may also improve the experiences of Indigenous patients receiving care from pharmacy professionals.

A variety of interrelated interpretations exist in terms of how Indigenous knowledge systems are defined. The United Nations Educational, Scientific and Cultural Organization (UNESCO)^12^ qualifies Indigenous knowledge as “the understandings, skills and philosophies developed by societies with long histories of interaction with their natural surroundings.” UNESCO has also noted that “[Indigenous] knowledge is integral to a cultural complex that also encompasses language, systems of classification, resource use practices, social interactions, rituals and spirituality.”^12^ Indigenous knowledge has also been defined as local knowledge that is held by Indigenous Peoples or local knowledge unique to a given culture or society.^13^ Indigenous knowledge systems correspond to the entire spectrum of philosophy, history, heritage, ethics, flora and fauna, educational processes and much more.^14^ Doaxter^15^ described Indigenous knowledge as “reasonable, deliberate and useful for making sense of life.” Similarly, Article 31 of the United Nations^16^ Declaration on the Rights of Indigenous Peoples (UNDRIP) described the right of Indigenous people to maintain, control, protect and develop their traditional knowledge.

Indigenous worldviews are often admired for the rich place-based oral histories that live within First Nations, Métis, Inuit and other Indigenous nations; however, in the Western scientific community of positivism and perceived objectivity, Indigenous worldviews are cast aside as mythical or even romantic ideas of the past and ways of being.^4,17^ Well known and widely regarded as a thought leader in Indigenous knowledge systems and worldview, Indigenous philosopher and historian Vine Deloria^4^ proposed that “there are literally millions of observed facts which simply do not appear in scientific writing because they would tend to raise doubts about the prevailing paradigm.” Seemingly, then, a fundamental difference between Western and Indigenous knowledge systems is whether such knowledge is captured and disseminated orally or in writing.

**Résumé**

- Il existe des différences significatives entre les visions du monde autochtone et occidentale.
- Chaque vision du monde est valable, importante et essentielle pour l’enseignement et la pratique de la pharmacie.
- Apprendre à connaître et à apprécier les tensions entre les visions du monde autochtone et occidentale peut améliorer l’expérience des professionnels de la pharmacie autochtone en matière de formation et de pratique, ainsi qu’améliorer l’expérience des patients autochtones qui reçoivent des soins de la part des professionnels de la pharmacie.

Deloria^4^ postulated that “the non-Western tribal equivalent of science is the oral tradition, the teachings that have been passed down from one generation to the next over uncounted centuries.” Such oral traditions (or “Indigenous science”) are composed of loosely held collections of anecdotal material that, when taken together, “explains the nature of the physical world as people have experienced it and the important events of their historical journey.”^4^ Deloria^4^ suggested that only 10% of the “information that Indigenous Peoples possess is presently in print and available for discussion. Without a traceable written history of Indigenous science, information possessed by Indigenous Peoples becomes valid only when offered by a white scholar recognized by the academic establishment; in effect, the colour of the skin guarantees scientific objectivity.”^4^ The Indigenous explanation, as Deloria^4^ described, “is always cast aside as a superstition, precluding Indians from having an acceptable status as human beings and reducing them in the eyes of educated people to a pre-human level of ignorance.” The survival of Indigenous Peoples for tens of thousands of years, based on each nation’s Indigenous knowledge, already legitimizes its content validity.^18^

In an Indigenous paradigm, knowledge is made available to Indigenous Peoples by visions and dreams, birds, animals, rivers and mountains, all of which are inaccessible to modern science.^4^ Atleo,^3^ too, described the acquisition of knowledge via the variety of visions that humans experience, whether in a nocturnal dream, waking dream, meditative vision, group vision or solitary vision, and highlighted the spiritual experience itself that results in the acquisition of knowledge in this way. Recognizing these important ways of knowing, knowledge is personal for non-Western peoples and impersonal for the Western scientist.^4^ Eurocentric Western people believe that anyone can use knowledge; for Indigenous Peoples, though, only those people given the knowledge by other entities can use it properly.^4^ Additionally, the major difference between Indigenous views of the physical world and Western science lies in the premise accepted by Indigenous Peoples and rejected by scientists: “The world in which we live is alive.”^4^

Frustratingly, sometimes the evolution and “modernization” of Indigenous knowledge is viewed as inauthentic. Atleo^3^ suggested that “change is not unusual to any culture or civilization, yet it is assumed that as soon as change is introduced to Indigenous cultures, they can no longer be authentic.” Inaccurately and unfairly, “authentic Indigenous cultures are thought to be those that have had no contact with the colonizing Westerner. It is a most arrogant position to hold because it attributes inordinate and unreasonable powers of transformation to the colonizers.”^3^ Like Western science and intellectual traditions, Indigenous knowledge is expected to evolve and grow, just as the places, spaces, animals, spirits and peoples who hold knowledge also evolve.

Beyond the acquisition of knowledge, its dissemination may also exist in culturally and tribal-specific ways. As Atleo^3^ highlighted, a Western scientist may work alone for years until a breakthrough is made, at which point the research outcome is disseminated through papers, publications and conferences, whereas an Indigenous knowledge-seeker may also spend years until something of great significance happens that affects the whole community, and only then is the outcome conveyed in songs, dances and appropriate ceremonial displays. Of course, Indigenous Peoples may (and do) also elect to disseminate knowledge in ways that are congruent with typical Western methods, such as papers, publications and conferences; however, often the paradigm from which the Indigenous researcher is coming will shine through in their use of storytelling in both written and oral dissemination methods, for example.

## Western intellectual traditions, science and knowledge systems

Describing Western intellectual tradition poses a significant challenge, as Western scientists do not have to repeatedly strive to validate themselves and their ways of knowing and are simply accepted by scholars and in the literature, which are grounded in Western intellectual thought and Western (or, non-Indigenous) knowledge systems. While innovation and novel ideas certainly are still presented in Western intellectual bodies, they are scrutinized, evaluated and disseminated by people and programs that are grounded in the same Western (non-Indigenous), positivistic and scientific worldviews. Even as a word, *science* privileges a very narrow meaning (the canonical Western science content taught in universities), which today can act in a neocolonizing way.^5^ The word *science* was “deliberately chosen in 1831 when some natural philosophers founded the British Association for the Advancement of Science and thereby professionalized natural philosophy into a new social institution, which they called ‘science’ for very political reasons.”^19^

Since the professionalization of natural philosophy into “science,” we have been socialized to interpret that Western science is superior as a result of the use of the scientific method, which is supposed to eliminate or at least drastically reduce the biases of the scientists. Deloria^4^ highlighted the obedience of scientists and scholars to the consensus opinions of their profession(s) and suggested this “usually means they pay homage to the opinions of scholars and scientists who occupy the prestige chairs at Ivy League and large research universities or even dead personalities of the past.” Deloria^4^ also offered a critical reflection regarding the evolution of science and its marginalizing nature, in that “institutionalization of science took many forms: the increasing tendency of people to look to scientists for reliable explanations about the world, the development of universities and colleges, sponsorship of scientific research by wealthy patrons and eventually the state.”

Clearly, science and the involvement in science was and still is a source of privilege, and attempts to disrupt its rigid structure are unlikely to be successful. Deloria^4^ highlighted the positivistic nature of Western intellectual traditions and scientific thought in the following statement: “We have been trained to believe that science is infallible in the sense that, while science does not know everything, its processes of investigation and experimentation are the best available so that, given time and resources, the truth will eventually be discovered.” Western intellectual traditions and the paradigm of science leave little, if any, room for multiple truths. When multiple truths exist, each truth is forever considered a theory until proven otherwise, using the scientific method, unlike Indigenous paradigms, which have space for multiple truths.^20^

The body of the Western scientific paradigm continues to be protected in peer-reviewed scholarly journals versus the oral history and land/place-based knowledge witnessed in Indigenous knowledge systems. Deloria^4^ argued that journals do not reflect science or human knowledge and instead “represent the subjects that are not prohibited in polite discussion by a few established personalities in the larger intellectual world.” He offered critical thoughts about Western science and the (dis)inclusion of Indigenous intellectual traditions by describing 2 things that need to be done before there can be any exchange of views between Indigenous Peoples and Western science. First, he noted that “corrective measures must be taken to eliminate scientific misconceptions about Indians, their culture and their past.”^4^ Second, there must be a way that “Indian traditions can contribute to the understanding of scientific beliefs at enough specific points so that the Indian traditions will be taken seriously as valid bodies of knowledge.”^4^ Both changes, however, involve a fundamental struggle over the question of authority, “since even when Indian ideas are demonstrated to be correct there is the racist propensity to argue that the Indian understanding was just an ad hoc lucky guess—which is perilously close to what now passes for scientific knowledge.”^4^ Despite such criticisms, however, Western intellectual traditions and Western science are critical for the effective and holistic education and practice of pharmacy.

## Conclusion

The profession of pharmacy has much to learn from Indigenous knowledge systems and the Indigenous paradigm. Without a concerted effort to learn, appreciate, understand and perhaps also apply Indigenous knowledge across the profession of pharmacy, the profession will continue to create and re-create neocolonial pressures on Indigenous Peoples both within and outside the profession. Pharmacy students and pharmacy practitioners are socialized into perceiving Western intellectual traditions as the superior knowledge system, and this socialization ostracizes and isolates Indigenous Peoples within the profession, as well as Indigenous patients and clients. Each knowledge system must be perceived as unique, valid and equally important in pharmacy education and practice. Like the braid of sweetgrass, each strand, or knowledge system, requires tension to be woven together into something larger and stronger in a way that allows for each strand to still maintain its own vital composition.

The ethnocentric positionality of Western intellectual traditions has impacted my own education, teaching and career trajectory as an Indigenous pharmacist and faculty member. For example, I have received verbal and written comments from students who have shared with me that there is “very little superiority in Indigenous medicine” and “the best argument for it is that it has been consistently used for hundreds and potentially thousands of years” (personal communications). To work toward an equality of knowledge systems in pharmacy education and practice, Western knowledge systems must continue to be interrogated in the same ways that other knowledge systems are scrutinized, which will aid in emphasizing that no single knowledge system is perfect or superior, but rather each school of thought is a contributor to, versus the entire basis of, our collective understanding and practice of pharmacy. Teaching, learning and practising pharmacy through only a Western lens contributes to much unfriendliness in the profession for Indigenous learners and practitioners who see and understand the world through an Indigenous worldview. As such, I will again propose that Western intellectual tradition may benefit from the use of an emulsifier, such as an Indigenous Western-trained pharmacist and educator. While this may be risky and challenging, I feel a strong sense of responsibility to contribute to reducing the (surface) tension.

To assist with the emulsification of Western and Indigenous knowledges in the context of pharmacy practice and education, I will offer 2 additional articles on some of the specific tensions that exist between these knowledge systems within the context of the profession of pharmacy. Identifying and analyzing such tensions will contribute to reconciling how we teach, learn and practise pharmacy, just as we have been called upon to do in the Truth and Reconciliation Commission of Canada’s calls to action.^21^ I invite you to join me in this series of articles as we expose the following tensions between Western and Indigenous worldview in the pharmacy profession: 1) what constitutes evidence and truth, 2) fragmentation and compartmentalization and 3) the lack of regard for land and spirituality.

*Kika-wāpamin mīnawā. Wā-nihšitotamank* (I will see you again. We are going to understand). ■
